# Inactivation of non-enveloped virus by 1,5 iodonaphthyl azide

**DOI:** 10.1186/s13104-015-1006-2

**Published:** 2015-02-15

**Authors:** Paridhi Gupta, Anuj Sharma, Viard Mathias, Yossef Raviv, Robert Blumenthal, Radha K Maheshwari

**Affiliations:** Department of Pathology, Uniformed Services University of the Health Sciences, Bethesda, MD USA; Basic Science Program, Leidos Biomedical Research, Inc., NCI Center for Cancer Research, Frederick National Laboratory for Cancer Research, Frederick, MD USA; Chemical Biology Lab, Center for Cancer Research, National Cancer Institute, Frederick, MD USA

**Keywords:** Inactivated, EMCV, Non-enveloped, Iodonaphthyl azide

## Abstract

**Background:**

A photoactive hydrophobic agent 1,5-iodonaphthyl-azide (INA), has been previously shown to completely inactivate the enveloped viruses. INA sequesters into the lipid bilayer of the virus envelope and upon UV-irradiation bind to the hydrophobic domains of the envelope glycoproteins. In our earlier study, we have shown that the Venezuelan equine encephalitis virus (VEEV) genomic RNA was also inactivated during the inactivation of the virus with INA.

**Findings:**

In the present study, we evaluated if the RNA inactivation property of INA can be used to inactivate non-enveloped RNA viruses. Encephalomyocarditis virus (EMCV) was used as a model non-enveloped virus. Treatment with INA followed by UV-irradiation resulted in complete inactivation of EMCV. RNA isolated from INA-inactivated EMCV was non-infectious and INA was found to be associated with the viral RNA genome. INA-inactivated EMCV induced robust total antibody response. However binding capacity of INA-inactivated EMCV to neutralizing antibody was inhibited.

**Conclusion:**

This is the first study to show that INA can completely inactivate non-enveloped virus. Our results suggest that the amino acid composition of the neutralizing epitope may interfere with the protective antibody response generated by the INA-inactivated non-enveloped virus.

**Electronic supplementary material:**

The online version of this article (doi:10.1186/s13104-015-1006-2) contains supplementary material, which is available to authorized users.

## Findings

Conventional methods of preparing chemically inactivated viral vaccines have several limitations such as denatured immunogens, short-lived immunity and, in some cases incomplete inactivation resulting in disease outbreaks in the vaccinees [1]. We and others have shown earlier that 1,5-iodonaphthyl-azide (INA), a photoactive hydrophobic alkylating compound, can inactivate enveloped viruses by covalently binding to the hydrophobic domains of the viral proteins present in the envelope lipid bilayer [2-11]. We also demonstrated that the infectious positive sense ssRNA viral genome of Venezuelan equine encephalitis virus (VEEV) was inactivated during inactivation by INA [5]. We hypothesize that in addition to the enveloped viruses, INA can also inactivate the non-enveloped viruses by inactivating the viral RNA genome and this property can be used to develop non-enveloped viral vaccine candidates. In this study, we used encephalomyocarditis virus (EMCV) as a non-enveloped virus model. EMCV is a *Cardiovirus* in the family *Picornaviridae* and like VEEV has a positive sense ssRNA genome. EMCV infects several animal species like pigs, rodents, cattle, elephants, non-human primates and humans and cause frequent outbreaks in the zoo animals [12-17].

EMCV was inactivated using INA (10 μM, 30 μM, 50 μM and 100 μM dose) and UV-irradiation, as described before [7]. Briefly, 500 μg of EMCV was passed through 30 gauge needle mounted on a 1 ml syringe. Samples were then mixed with desired dose of INA and incubated for 30 min in the dark at room temperature. Samples were centrifuged at 1000 rpm for 1 min to remove precipitated INA crystals. Supernatant containing the virus suspension was transferred to a new 1.5 ml clear wall tube and irradiated for 5 min using 100 W mercury UV lamp (Osram Sylvania Products Inc., Winchester, KY and UVP, LLC, Upland, CA) with intermittent vortexing using the following setup: A clear glass plate filter was placed immediately in front of the lamp to filter out the short wavelength UV and allow transmission of the longer wavelengths of UV light. A water filter was placed at a distance of 6–7 cm from the UV lamp to prevent heating of the samples and the samples were placed 6–7 cm away from the water filter. A similar set up delivered a UV dose of 10 mW/cm^2^.s in the earlier studies [4,9,11]. The following control and test groups were taken: **Control samples:** (1) PBS only **(UN),** (2) EMCV only **(E),** (3) EMCV plus UV-irradiation **(Ei),** (4) EMCV plus 1% DMSO **(ED),** (5) EMCV plus 1% DMSO plus UV-irradiation **(EDi)**. INA was dissolved in DMSO, therefore, the maximum concentration of DMSO (1%) achieved with 100 μM INA dose was used as control. **Test samples:** (1) EMCV plus INA (at 10 μM, 30 μM, 50 μM and 100 μM doses of INA and referred as **EI**_**10,**_**EI**_**30,**_**EI**_**50**_**and EI**_**100**_, respectively) and (2) EMCV plus INA plus UV-irradiation (referred as **EI**_**10**_**i, EI**_**30**_**i**_**,**_**EI**_**50**_**i and EI**_**100**_**i**, respectively). Inactivation of the virus was assessed by the combined results of cytopathic effect (CPE), virus titer in cell supernatants, and EMCV-3D gene (encoding for the viral polymerase) specific RT-PCR on total cellular RNA isolated from the infected cell (Forward primer- 5′ TCCCGTTTGCGGCAGAAAGATT 3′; Reverse primer- 5′ AAGCGGAACATTGCCACCGAAT 3′).

**Figure 1:**
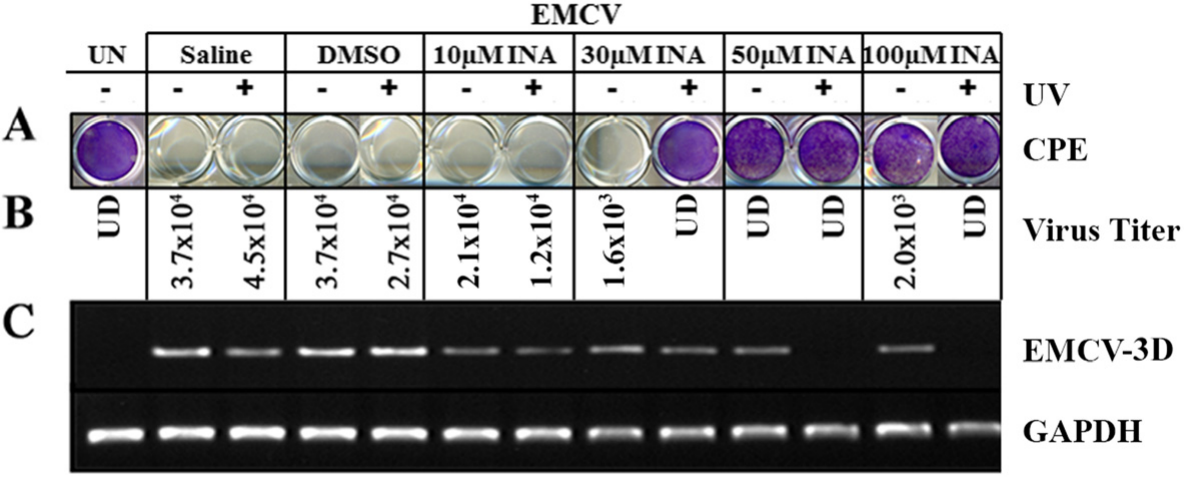
**Inactivation of EMCV by INA. A)** L-cells were infected with virus preparations at an MOI = 10. At 72 h post infection, cells were fixed and stained with crystal violet. The wells with live cells are stained in blue. Clear wells indicate cell death due to virus infection. **B)** Virus titer (represented as TCID_50_/ml) was measured in the supernatant of L-cells infected with control and test samples. The results are representative of at least 4 replicates. **C)** EMCV-3D gene specific RT-PCR was done on RNAs isolated from cells infected with controls or test EMCV samples. GAPDH was used as the reference housekeeping gene. UN: Uninfected cells, UD: under the detection limit, UV: Ultraviolet rays.

INA inactivated EMCV and a complete loss of EMCV infectivity was achieved at 50 and 100 μM dose of INA combined with the UV-irradiation (Figure 1). 30 μM INA in combination with UV-irradiation partially inhibited EMCV infectivity. Treatment with INA alone at 50 and 100 μM doses also partially inhibited the infectivity of EMCV (Figure 1A and B). Inhibition of EMCV infectivity by INA alone or in combination with UV-irradiation, in CPE and virus titer assays, may have been observed due to the limit of detection of virus in these assays. Therefore, a more sensitive RT-PCR assay for EMCV 3D-gene was used, which showed that complete inactivation of EMCV occurred only at 50 and 100 μM dose of INA in combination with UV-irradiation (Figure 1C). Data suggests that INA inactivation of EMCV may be dose dependent, but is not conclusive. Partial inhibition of EMCV by INA alone observed in this study was different from our earlier studies with enveloped viruses where no adverse effect of INA alone was observed on VEEV and CHIKV [5-7]. As both EI_50_i and EI_100_i showed complete inactivation, only EI_100_i was used in the remaining experiments.

**Figure 2:**
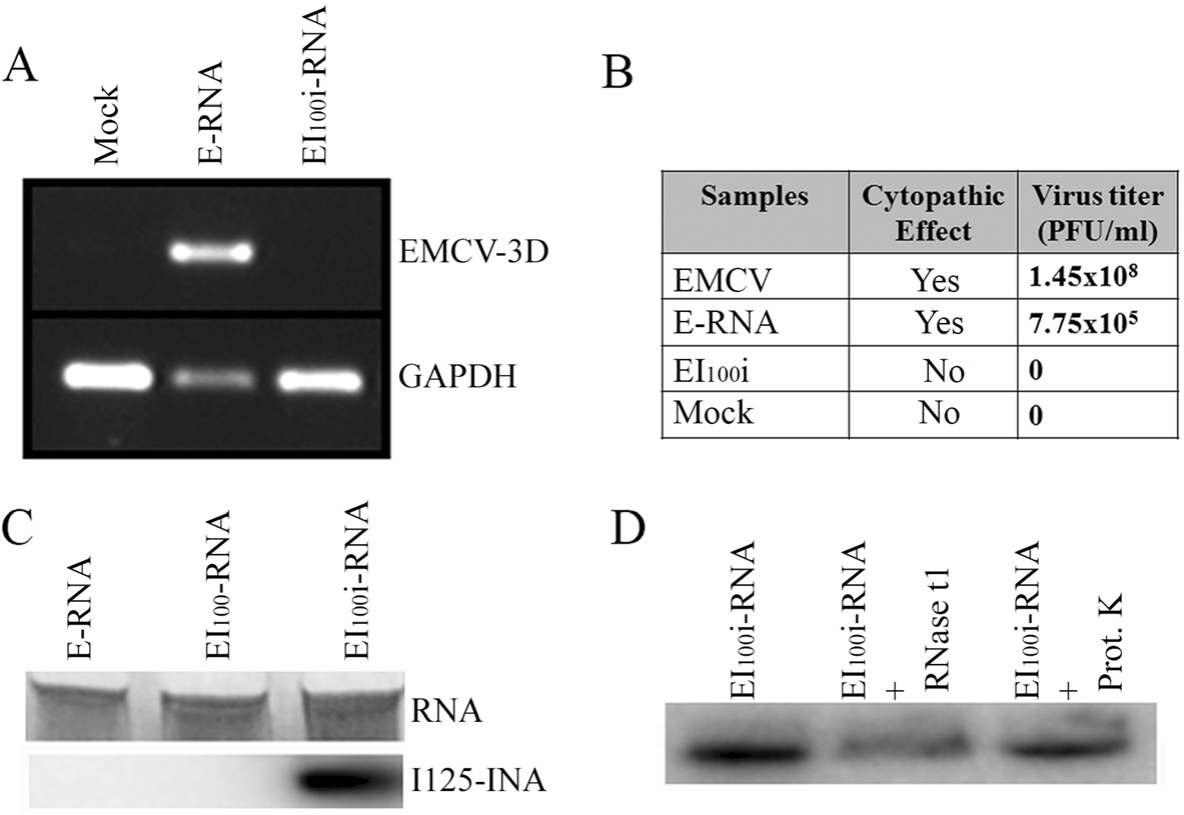
**INA binds to the viral RNA and renders it non-infectious** ***in-vitro*** **. (A)** L-cells were transfected with RNA isolated from infectious EMCV (E-RNA) or EI_100_i (EI_100_i-RNA) or mock transfected. EMCV-3D gene specific RT-PCR was done on the total RNA isolated from cells after 48 h post transfection to test for EMCV replication. **(B)** Virus replication in cell supernatants was measured by standard plaque assay. No virus replication was detected in the supernatant of the cells transfected with the EI_100_i-RNA. Virus titer in cells transfected with E-RNA was similar to the samples infected with control live EMCV virus. **C)** RNA was isolated from EMCV (E-RNA), EMCV treated with 100 μM I125-labeled INA (EI_100_-RNA), and EMCV treated with 100 μM I125-labeled INA in combination with UV (EI_100_i-RNA). Co-localization of INA with RNA was tested by overlapping silver staining of RNA (upper panel) with autoradiograph of I125-labeled INA (lower panel). **D)** RNA isolated from EI_100_i (EI_100_i-RNA) was treated with RNase t1 or Proteinase K to test the binding of INA to EMCV RNA.

To evaluate the effect of INA-inactivation on EMCV genome infectivity, RNA was isolated from untreated infectious- EMCV (E-RNA) and EI_100_i (EI_100_i-RNA) using the Viral RNA/DNA purification kit (Life Technologies Inc., Carlsbad, CA). L cell monolayers were transfected with 100 ng RNA mixed with 3 μl Fugene HD transfection reagent (Roche Applied Sciences, Indianapolis, IN) and 97 μl optiMEM as per manufacturer’s protocol. Virus replication in the transfected cells was evaluated by EMCV 3D-gene expression using a specific RT-PCR on the RNA isolated from the cells at 48 h post transfection. No virus specific amplification was observed in the cells transfected with EI_100_i-RNA unlike those transfected with E-RNA (Figure 2A). Similar results were also observed in a parallel experiment, where virus replication was evaluated by plaque assay in the cell supernatants at 72 h post transfection (Figure 2B).

To evaluate whether INA directly interacts with the viral RNA genome during the inactivation process, autoradiography was performed with the RNA isolated from EMCV inactivated with 100 μM of I125-labeled INA. Virus particles were treated with RNase free DNase (Promega corp, Madison, WI) before RNA isolation to eliminate any cellular DNA contamination. RNA samples (500 ng) were then subjected to electrophoresis on 6% TBE-urea gel followed by silver staining according to the manufacturer’s instructions (Silver stain kit, Life Technologies Inc., Carlsbad, CA). Gel was exposed to the X-ray film to detect I125-labeled INA specific band and its association with the viral RNA. INA specific bands were only obtained and co-localized with RNA isolated from EMCV sample treated with INA in combination with UV-irradiation (EI_100_i-RNA, Figure 2C). No such co-localization was observed with the RNA isolated from EMCV treated with INA alone (EI_100_-RNA, Figure 2C). EMCV RNA is present in complex with the VPg protein, which is present at the 5′ end of the viral genome [18]. To check whether INA is binding to the VPg protein or to the viral RNA genome, RNA isolated from the virus preparations were treated with RNase t1 or proteinase K at 37°C for 1 hr. Treatment with proteinase K resulted in slight reduction in the I125 labeled-INA specific band intensity, whereas, treatment with RNase t1 resulted in greater loss in the band intensity (Figure-2D) indicating the association of INA with viral RNA. Taken together, these results suggest that although some of the INA may bind to the viral VPg protein; a larger percentage of INA binds to the viral RNA genome and this binding is dependent on UV-irradiation.

Safety and protective efficacy of EI_100_i against lethal challenge was also evaluated *in-vivo* under two pilot studies as described in Additional file 1: Figure S1A & D. All experiments were conducted in accordance with the Guide for the Care and Use of Laboratory Animals (Committee on Care And Use of Laboratory Animals of The Institute of Laboratory Animal Resources, National Research Council, NIH Publication No. 86-23, revised 1996) and approved by the Uniformed Services University of the Health Sciences (USUHS) institutional animal care and use committee (IACUC). ***Study-1:*** 4-5 week old male CD-1 mice were immunized intraperitoneally (i.p.) with saline (n = 5) or 10^8^ plaque forming unit (PFU) of EI_100_i (n = 10) on Day 0 and Day 14. PFU for inactivated virus was extrapolated from the PFU value of the live EMCV stock that was used for the inactivation. Animals were challenged with 10^8^ PFU of infectious EMCV through intra-peritoneal (i.p.) route on Day 28 (Additional file 1: Figure S1A). ***Study-2***: CD-1 male mice (4-5 weeks old) were immunized with saline (n = 4) or 10^8^ PFU of EI_100_i (n = 6) or EI_100_i mixed with equal volumes of adjuvant Alhydrogel® (Alum; final Al concentration of 2 mg/ml; Brenntag Biosector, Fredeikssund, Denmark) (n = 6) on Day 0, Day 14 and Day 28. Mice were challenged with 2×10^7^ PFU of virulent EMCV through i.p. route on Day 63 (Additional file 1: Figure S1D). Animals were monitored for clinical signs of disease such as weight loss, ruffled fur, hunched back, lethargy and paralysis and were euthanized when found moribund. All animals that received the immunization with EI_100_i developed normally without any clinical symptoms of disease and gained weight similar to that of the animals in saline control group (Additional file 1: Figures S1B & E). A robust total IgG response was observed post immunization indicating highly immunogenic nature of EI_100_i (Additional file 1: Figures S1C & F). Surprisingly, irrespective of the robust antibody response immunization with EI_100_i failed to protect the animals against infectious EMCV challenge (Additional file 2: Table-S1).

**Figure 3:**
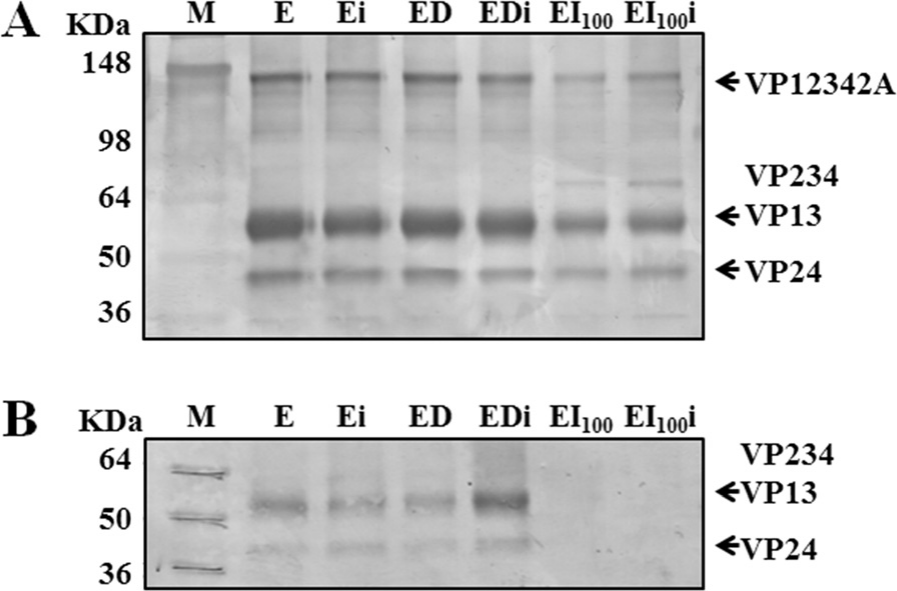
**Antigenicity of INA-inactivated EMCV. A)** Western blot analysis using polyclonal anti-EMCV antibody. **B)** Western blot analysis using polyclonal neutralizing anti-EMCV antibody.

To test if inactivation with INA interfered with the antigenicity of EMCV, binding of EI_100_i to anti-EMCV antibody was evaluated. Western blot analysis using a polyclonal anti-EMCV antibody (1:100; EMCV antiserum, Catalog#301-MDV, USDA) revealed four major bands corresponding to EMCV structural proteins in the test and the control samples (Figure 3A). Further analysis using a polyclonal neutralizing anti-EMCV antibody (1:50; Catalog#315-MDV, USDA APHIS, NVSL, Ames, IA), however, revealed complete loss of antibody binding capacity of EI_100_ and EI_100_i samples (Figure-3B). Reduction in the antibody binding capacity after inactivation with INA has also been reported with enveloped viruses [6,7,11]. INA is known to bind to cysteine molecules in a peptide [2,3]. Neutralizing epitope on the capsid protein, VP1, of EMCV contains 5 cysteine residues [18,19]. We hypothesize that binding of INA to these cysteine residues may have resulted in masking of the protective epitopes present on the surface of EMCV. This would explain the complete loss of binding to the neutralizing antibodies and failure of EI_100_i to protect against challenge with infectious EMCV. Complete loss of binding of EMCV treated with INA alone (EI_100_; Figure 3B) was unexpected as INA in absence of UV has not been reported to bind to the viral proteins [2]. This suggests either a non-specific interaction of INA with viral proteins or interaction via an unknown mechanism and will need further evaluation.

This is the first study to demonstrate the inactivation of non-enveloped virus by INA and it’s binding to the viral genomic RNA, which was dependent on UV-irradiation. Though the mechanism by which INA binding results in inactivation of viral RNA is not known, it may be possible that INA interferes with the interaction between the viral genomic RNA and the replication complex during the virus replication process. Since, INA seems to inactivate viruses by two independent mechanism *i.e.*, targeting viral envelope proteins [2-4] and viral genomic RNA; this method presents a novel inactivation strategy for developing second generation inactivated virus vaccine candidates for both the enveloped and the non-enveloped viruses. However, its application may be limited by the non-specific interaction of INA with the viral proteins, as observed in this study. Application of INA inactivation method may also be limited for certain viruses where the protective epitopes consist largely of cysteine residues and should be considered while using INA for inactivated viral vaccine development.

## Additional files

Additional file 1: Figure S1.
*In-vivo* evaluation of EI_100_i as vaccine candidate. Two separate studies were conducted to evaluate the immunization efficacy and protective response of EI_100_i against infectious EMCV challenge. In the first study, no adjuvant was use and two immunizations were done two weeks apart **(A)**. In the second study, adjuvant Alum was used and three immunizations, each two weeks apart, were done **(D)**. The body weight of the animals was monitored weekly till the end of study 1 **(B)** and study 2 **(E)**. Animals were bled at pre-determined time points to evaluate the seroconversion of the animals post immunization. The total IgG response against EMCV was evaluated by the end-point dilution method in the serum collected at day 13 and 27 for the study 1 **(C)** and day 13, 27 and 42 for the study 2 **(F)**. Pre-bled (day -3) and saline administered mouse serum were used as negative controls. End point titers were determined at an absorbance greater than or equal to the mean absorbance for negative controls plus three times the standard deviation. A significant increase in the total IgG levels was observed after first immunization and each booster dose in both the studies. No significant increase in the total antibody was observed in the presence of Alum. Two tail student’s *t*-test was used to calculate the significance (p-value: * ≤ 0.01, ** ≤ 0.001, *** ≤ 0.0001). Significance in comparison to the control group is also indicated (^§^ p-value ≤ 0.01). (TIFF 715 kb) Additional file 2: Table S1. Protection after immunization with EI_100_i.

## Abbreviations

CPE: Cytopathic effect
DMSO: Dimethyl sulfoxide
EMCV: Encephalomyocarditis virus
E: EMCV only
Ei: EMCV plus UV-irradiation
ED: EMCV plus 1% DMSO
EDi: EMCV plus 1% DMSO plus UV-irradiation
EI_10_: EI_30_, EI_50_ and EI_100_: EMCV plus 10 μM, 30 μM, 50 μM and 100 μM dose of INA, respectively
EI_10_i: EI_30_i, EI_50_i and EI_100_i: EMCV plus 10 μM, 30 μM, 50 μM and 100 μM dose of INA, respectively, plus UV-irradiation
E-RNA: RNA isolated from EMCV
EI100-RNA: RNA isolated from EMCV
EI_100_i-RNA: RNA isolated from EMCV inactivated with 100 μM dose of INA and UV-irradiation
GAPDH: Glyceraldehyde 3-phosphate dehydrogenase
IACUC: Institutional Animal Care and Use Committee
INA: 1,5 iodonaphthyl azide
PBS: Phosphate buffered saline
PFU: Plaque forming unit
RT-PCR: Reverse transcription-Polymerase chain reaction
UN: Uninfected
USUHS: Uniformed Services University of the Health Sciences
UV: Ultraviolet
VEEV: Venezuelan equine encephalitis virus
