# Supplementary figures and images for: Inactivation of non-enveloped virus by 1,5 iodonaphthyl azide
Source: BMC Res Notes. 2015 Feb 15;8:44. doi: 10.1186/s13104-015-1006-2 (PMC4339248; doi:10.1186/s13104-015-1006-2)

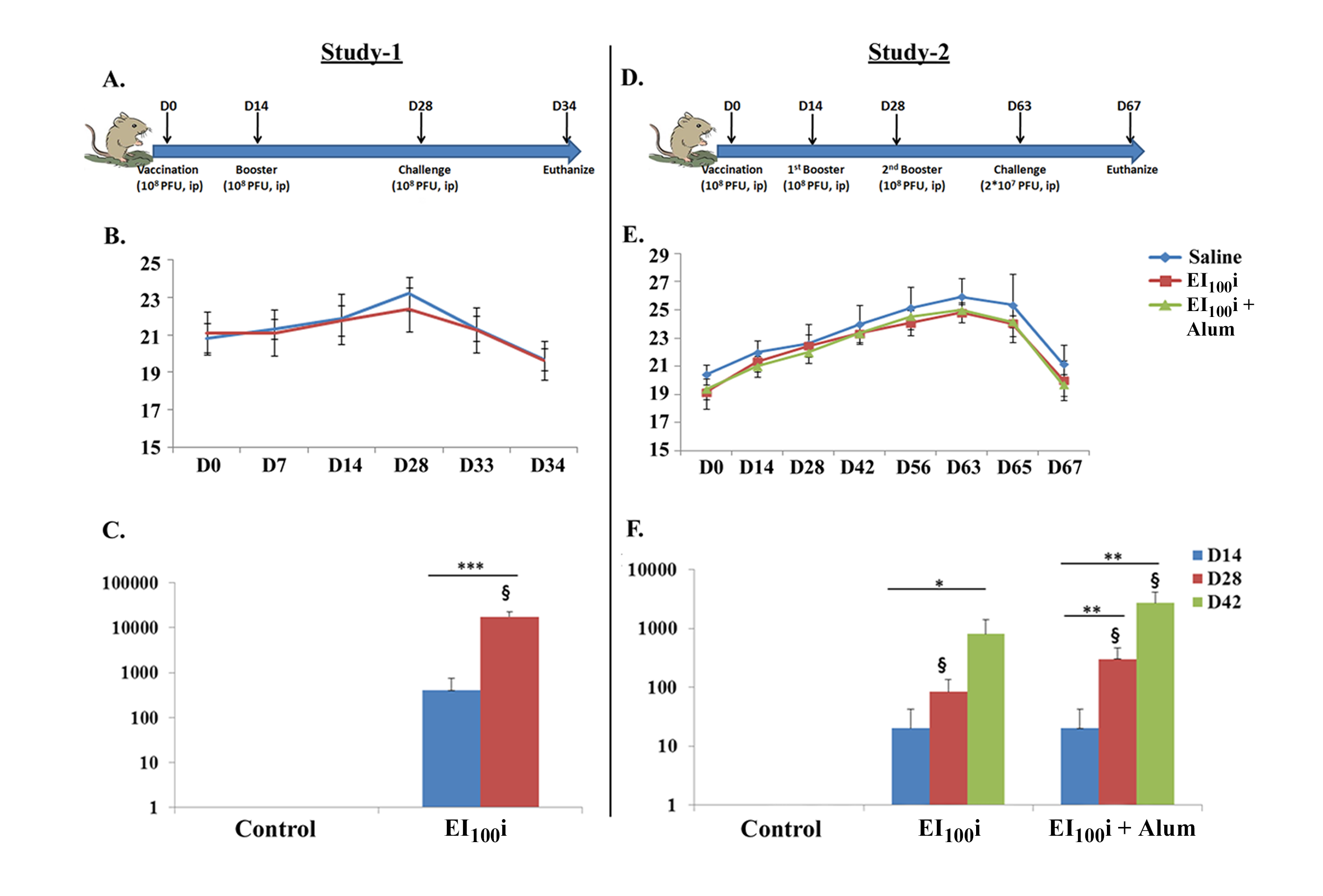

Supplement: Additional file 1: Figure S1. — In-vivo evaluation of EI100i as vaccine candidate. Two separate studies were conducted to evaluate the immunization efficacy and protective response of EI100i against infectious EMCV challenge. In the first study, no adjuvant was use and two immunizations were done two weeks apart (A). In the second study, adjuvant Alum was used and three immunizations, each two weeks apart, were done (D). The body weight of the animals was monitored weekly till the end of study 1 (B) and study 2 (E). Animals were bled at pre-determined time points to evaluate the seroconversion of the animals post immunization. The total IgG response against EMCV was evaluated by the end-point dilution method in the serum collected at day 13 and 27 for the study 1 (C) and day 13, 27 and 42 for the study 2 (F). Pre-bled (day -3) and saline administered mouse serum were used as negative controls. End point titers were determined at an absorbance greater than or equal to the mean absorbance for negative controls plus three times the standard deviation. A significant increase in the total IgG levels was observed after first immunization and each booster dose in both the studies. No significant increase in the total antibody was observed in the presence of Alum. Two tail student’s t-test was used to calculate the significance (p-value: * ≤ 0.01, ** ≤ 0.001, *** ≤ 0.0001). Significance in comparison to the control group is also indicated (§ p-value ≤ 0.01). (TIFF 715 kb) [file 13104_2015_1006_MOESM1_ESM.tiff]
