# Supplementary material for: Inactivation of non-enveloped virus by 1,5 iodonaphthyl azide
Source: BMC Res Notes. 2015 Feb 15;8:44. doi: 10.1186/s13104-015-1006-2 (PMC4339248; doi:10.1186/s13104-015-1006-2)
Supplement: Additional file 2: Table S1. — Protection after immunization with EI100i. [file 13104_2015_1006_MOESM2_ESM.docx]

**Supplementary table-1:** Protection after immunization with EI_100_i.

|  | **Study-1** | | **Study-2** | | |
| --- | --- | --- | --- | --- | --- |
|  | **Saline** | **EI_100_i** | **Saline** | **EI_100_i** | **EI_100_i + Alum** |
| **EMCV Challenge dose** | **1x10^8^** | **1x10^8^** | **2x10^7^** | **2x10^7^** | **2x10^7^** |
| **Number of animals/group** | **5** | **10** | **4** | **5** | **6** |
| **Mean survival time (Days)** | **3.0** | **3.0** | **4.5** | **4.6** | **4.8** |
| **Percent Protection** | **0** | **0** | **0** | **0** | **0** |

Groups of mice were immunized with EI_100_i followed by virulent virus challenge in two different studies (Supplementary Figure-1A and D). No protection was observed upon immunization in either of the studies.
